# Viruses Contained in Droplets Applied on Warmed Surface Are Rapidly Inactivated

**DOI:** 10.1264/jsme2.ME14108

**Published:** 2014-12-03

**Authors:** Swan Firquet, Sophie Beaujard, Pierre-Emmanuel Lobert, Famara Sané, Delphine Caloone, Daniel Izard, Didier Hober

**Affiliations:** 1 Université Lille 2, Faculté de Médecine, CHRU Lille, Laboratoire de Virologie EA3610, Loos-Lez-Lille 59120, France; 2 CHRU Lille Laboratoire de Bactériologie, Lille 59037, France

**Keywords:** heat, minute mouse virus, coxsackievirus B4, influenza virus type A, herpes simplex virus type 1

## Abstract

Heat inactivation of viruses was reported, however, the thermal resistance of viruses in droplets has not been studied. The aim of this study was to evaluate the pattern of heat resistance of minute virus of mice (MVM), coxsackievirus B4 (CVB4), influenza A virus (H1N1), and herpes simplex virus type 1 (HSV1) contained in droplets. Four μL droplets containing viruses (> 10^4.5^ TCID_50_) were applied onto warmed surface obtained by using a self-made heating device. Viral suspensions were exposed to temperatures ranging from 70 to 130°C for 0 to 90 min depending on the virus, and then the recovered viral preparations were tittered. Inactivation rates were calculated from curves that were analysed according to the first order kinetics model. Full inactivation was obtained for MVM in 90 min at 80°C and in 2 s at 130°C, for H1N1 in 14 s at 70°C and in 1 s at 110°C, for CVB4 and HSV-1 in 5 s and 7 s respectively at 70°C and in 1 s at 100°C. Clearly, MVM was more resistant than H1N1 that was more resistant than HSV-1 and CVB4, which was reflected by increasing inactivation rates. The impact of short time exposure to heat onto the infectivity of viruses contained in a small volume of suspension has been determined. For the first time, the inactivation of viral particles contained in drops exposed to temperatures higher than 100°C has been investigated. It appears that heating can have an unexpected faster virucidal effect than previously described.

Viruses can be transmitted through aerosol or droplet. Therefore airfilters are used to collect viruses (12). However, the filtered viruses remain infectious and thus they can cause infection through contact or reaerosolization (4, 22). Various virucidal processes to disinfect airfilters have been reported (17, 20, 21, 26). Thermal inactivation of viruses has been described (1) and recently, infrared (IR) radiation heating was used to disinfect filters which trapped micro-organisms (8).

In the present study, the virucidal effect of warmed surface on viruses contained in droplets has been investigated. A system has been developed to evaluate the resistance to heating of non-enveloped viruses; minute mouse virus (MVM), coxsackievirus B4 (CVB4), and two enveloped viruses, influenza virus type A (H1N1) and herpes simplex virus type 1 (HSV-1). These viruses are relevant models to assess the virucidal effect of heat. MVM is a parvovirus, which is known to be the most resistant to thermal inactivation (2), and the other viruses can be responsible for nosocomial infections (3, 16), and for each virus, infectious titers higher than 10^6^ TCID_50_ mL^−1^ can be obtained *in vitro*.

## Materials and Methods

### Virus and Cell lines

CVB4 E2 is a strain provided by Ji-Won Yoon, Julia McFarlane Diabetes Research Center (Calgary, Alta., Canada) (14). The viruses were propagated in a flask on the appropriated cell lines: HSV-1 (ATCC VR-260) on Vero (ATCC CCL-81) cells, CVB4 on Hep-2 (ATCC CCL-23) cells, MVM (ATCC VR-1346) on A9 (EACC N° 85011426) cells and H1N1 A/PR/8/34 (ATCC VR-1469) on MDCK (NBL2) (ATCC CCL-34) cells. Infected Vero and Hep-2 cells were cultured in supplemented Eagle’s essential medium (MEM; Invitrogen, France) and Infected A9 cells in supplemented Dulbecco’s modified Eagle medium (DMEM). MEM and DMEM media were supplemented with 2% fetal bovine serum (FBS), 1% non-essential amino acids and 1% L-glutamine at 37°C in a 5% CO2 atmosphere. Infected MDCK cells were cultured in MEM at 35°C in a 5% CO2 atmosphere. When a cytopathic effect of at least 75% appeared, the cells were scratched and viral particles were released by three freeze–thaw cycles; after centrifugation at 2,000 g for 10 min at 4°C the supernatants were harvested and afterwards they were aliquoted and stored at −80°C. In addition to CVB4 stock obtained from infected-Hep-2 cell cultures as stated above, CVB4 has been purified as previously described by our team with modifications (6). Briefly clarified supernatant of CVB4-infected Hep-2 cell culture lysates was pelleted by using PEG centrifugation at 8,000 g for 20 min at 4°C, and then ultracentrifuged onto CsCl layers. Afterwards CVB4 was desalted and resuspended in PBS and aliquots were stored frozen at −80°C.

### Virus titration

Fluids were distributed in six replicates in 96-well plates and serially diluted from 10^−1^ to 10^−8^ in MEM supplemented with 2% FBS, 1% non-essential amino acids and 1% L-glutamine for HSV-1 and CVB4; DMEM supplemented with 2% FBS and 1% L-glutamine for MVM; and MEM for H1N1. Then the plates were incubated for 5 d (H1N1 and CVB4), 7 d (HSV-1), 3 weeks (MVM) in a 5% CO_2_ atmosphere at 37°C. Afterwards the plates were examined using an inverted microscope to evaluate the extent of the virus-induced cytopathic effect in the cell culture. Calculation of estimated virus concentration was carried out by the Spearman-Kärber method and expressed as log_10_ TCID_50_ 4 μL^−1^ (10).

### Apparatus and heat inactivation

The apparatus was composed by a hotplate which heated a glycerol bath in a becher. Temperature was checked by electronic thermometer (AVAX, UK). An aluminium cone was plunged into hot glycerol. After a few seconds, 4 μL of virus suspension were put on aluminium surface during desired time. 200 μL of cool titration media were then quickly added and withdrawn, and then mixed with 800 μL of cool titration media.

### RT Real-Time PCR

CVB4E2 positive strand RNA was quantitated by two-step quantitative RT-PCR as described previously (25). Briefly, total RNA was extracted with RNeasy minikit (Qiagen, Valencia, Calif.) and resuspended. Total RNA was measured by a quantitative RT-QPCR for RNA with the Affinityscript QPCR cDNA synthesis kit and the brilliant II QPCR kit (Agilent technologie stratagene). Positive strand specific RT was carried out on extracted RNA by using the reverse primer at 42°C for 15 min. PCR was performed with universal cycle conditions (10 min at 95°C, 40 cycles of 30 s at 60°C) on a Mx3000p (stratagene). The following primers, used to detect CVB4E2 RNA, were located within the enterovirus 5′-nontranslated region, which is highly conserved among enterovirus serotypes: CVB4 forward (5′-CCC TGA ATG GGG CTA ATC) and CVB4 reverse (5′-ATT GTC ACC ATA AGC AGC CA). The sequence of the CVB4 probe was 5′-VIC-AAC CGA CTA CTT TGG GTG TCC GTG TTT-TAMRA (Applied Biosystems). The absence of contaminating DNA in samples was checked by RT-PCR without the reverse transcriptase enzyme. Primers and probe pairs were designed with PrimerExpress software, and the data were analyzed with Sequence Detector version 1.6.3 (both from Perkin-Elmer, Boston, Mass. Results were expressed as cycle threshold (Ct) which is inversely proportionate to RNA level.

### Modeling of inactivation

The first-order kinetic model assumes a linear relationship between the decreases in logarithmic reduction of the number of survivors over treatment time according to the formula

log (S[t])=kt

S(t) is the ratio between the infectious titer after an exposure time t, *i.e.*, N(t) (TCID_50_ 4 μL^−1^), and the initial infectious titer N_0_ (TCID_50_ 4 μL^−1^). K is the inactivation rate in log_10_ TCID_50_ 4 μL^−1^ s^−1^, and t is the treatment time (seconds).

### Statistical Analysis

Statistical analysis of the results was performed by Mann–Whitney when appropriate. Curves were compared using an analysis of covariance (ANCOVA) when appropriate. Differences were considered to be statistically significant when *P* < 0.05. Statistical analysis was performed using Graphpad Prism version 5.00 (Graphpad Software, San Diego, USA).

## Results

Virus inocula (4 μL) were applied in cone shaped aluminium foils that were set in a glycerol bath at various temperatures (70, 80, 90, 100, 110, 120, 130°C). 1 s to 90 min after inoculation, 200 μL of cool media were added and afterwards harvested to recover the virus inocula. Harvested fluids were spiked to 800 μL of cool media and then titration was carried out.

**Fig. 1:**
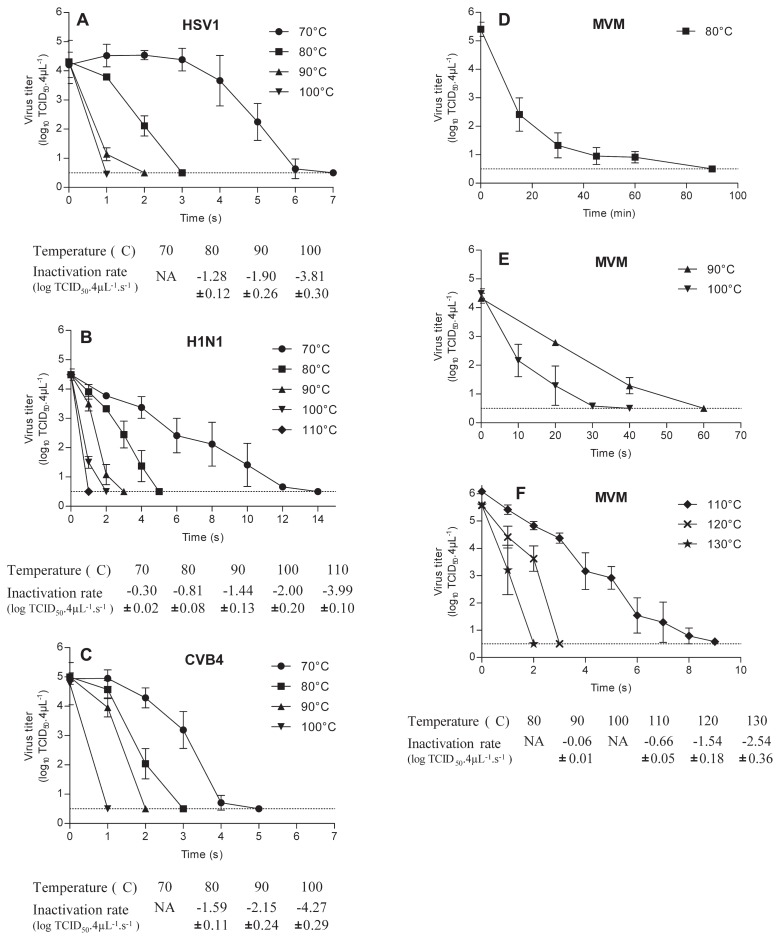
Viruses exposed to heat. 4 μL of culture supernatant fluids containing H1N1 (A), CVB4 (B), HSV-1 (C), or MVM (D, E, F) were applied on aluminium cones which were plunged into hot glycerol. Thereafter heated inocula were recovered using 200 μL of cool titration media which were then added to 800 μL of cool titration media and the infectious titers were determined and expressed as log_10_ TCID_50_ 4 μL^−1^. Inactivation rate for the survival curves of H1N1, CVB4, HSV-1, and MVM, expressed as log_10_ TCID_50_ 4 μL^−1^ s^−1^, are shown in tables below graphs. NA: represents curves that do not match with first-order kinetic model. Dashed line represents detection limit of the test. The results are the mean ± SD of four independent experiments.

When drops containing enveloped viruses, HSV1 and H1N1 were heated, the pattern of data was similar; however, H1N1 was more resistant to inactivation by heating compared with HSV1. Indeed, at 70°C the titer of HSV1 was reduced to 0.63 log_10_ TCID_50_ 4 μL^−1^ in 6 s, while, in these conditions, the titer of H1N1 was 2.42 log_10_ TCID_50_ 4 μL^−1^ (*P*=0.017) (Fig. 1). A 4 log_10_ reduction of infectious titer of drops containing H1N1 and HSV was obtained respectively, in less than 5 s and 3 s at 80°C, 3 s and 2 s at 90°C, 2 s and 1 s at 100°C.

The inactivation rates of H1N1 vs HSV1 were, respectively, −0.81 vs −1.28 log_10_ TCID_50_ 4 μL^−1^ s^−1^ (*P*<0.0001) at 80°C, −1.44 vs −1.90 log_10_ TCID_50_ 4 μL^−1^ s^−1^ (*P*<0.0001) at 90°C, −2.00 vs −1.77 log_10_ TCID_50_ 4 μL^−1^ s^−1^ (*P*<0.0001) at 100°C.

Drops containing CVB4 a non-enveloped virus, were inactivated with kinetics roughly similar to those of HSV1. Compared with H1N1, CVB4 appeared to be less resistant to inactivation by heating. Indeed CVB4 was dramatically inactivated, −4.23 log_10_ TCID_50_ 4 μL^−1^, at 70°C in 4 s whereas in similar conditions the inactivation of H1N1 was only −1.13 log_10_ TCID_50_ 4 μL^−1^ (*P*=0.028). Inactivation rates of CVB4 vs H1N1, at 80°C, 90°C, and 100°C were, respectively, −1.59 vs −0.81 (*P*<0.0001), −2.15 vs −1.44 (*P*<0.0001), and, −4.27 vs −2.00 log_10_ TCID_50_ 4 μL^−1^ s^−1^ (*P*<0.0001).

The impact of temperatures less than or equal to 100°C on to the infectious level of MVM contained in 4 μL of viral suspension was moderate, since an inactivation (up to 4 log_10_) was obtained only when drops were exposed to 80°C for 30 min, 90°C for 60 s (inactivation rate: −0.06 log_10_ TCID_50_ 4 μL^−1^ s^−1^) and 100°C for 30 s. Temperatures higher than 100°C, 110°C, 120°C and 130°C for 9, 3 and 2 s respectively resulted in a dramatic inactivation (> 4 log_10_).

In so far as it was reported that metals, like silver or copper, are able to inactivate viruses (6, 14, 17) we investigated, in the CVB4 model, whether aluminium foil used in our system was responsible for viral inactivation. Medium with or without aluminium flakes was heated at 90°C for 30 s before cooling at 4°C and then mixed with CVB4 suspensions (8.5 log_10_ TCID_50_ mL^−1^). The mixtures were incubated, at room temperature, for 30 min afterwards the level of infectious particles were determined. The level of infectious particles was not reduced when the viral suspension was incubated in medium containing aluminium (data not shown).

**Fig. 2:**
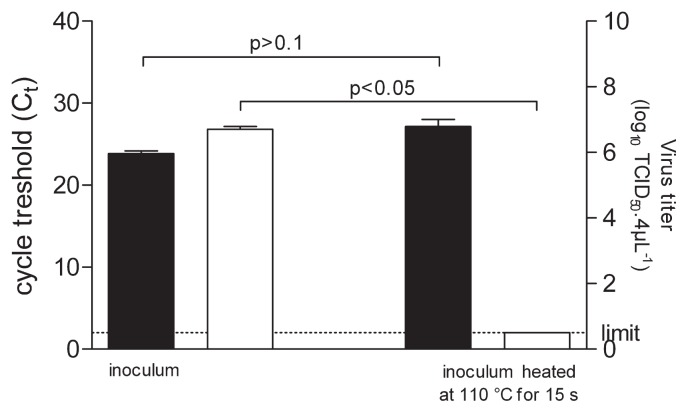
Quantification of CVB4 RNA and level of infectious particles. 4 μL of culture supernatant fluid containing CVB4 were applied on aluminium foil in quadruplicate. Inocula on aluminium foil were treated 15 s at 110°C, and thereafter recovered. The infectious titers of inocula were determined and expressed as log_10_ TCID_50_ 4 μL^−1^ (■). RNA was extracted from the harvest fluid and the level of viral RNA was measured by quantitative RT-PCR and expressed as C_t_ (□). The results are the mean + SD of four independent experiments.

Reduction of infectious titers in recovered inocula, when drops were heated on aluminium foil, raised several issues. The efficiency of recovering viral particles in these conditions was questioned. Therefore, on the one hand the infectious titer of CVB4 was determined and on the other hand, RNA was extracted to measure the amount of viral RNA by RT real-time PCR in order to estimate the level of viral particles. The amount of viral RNA in drops heated at 110°C for 15 s and in controls was closely similar as displayed by the pattern of Ct values (Ct = 23.86 vs 27.15, respectively) whereas the infectious titer values were markedly different (6.21 log_10_ reduction) (Fig. 2). These data brought evidence that heated viral particles were readily recovered and that heating reduced the infectious level of drops containing CVB4 heated on aluminium foil surface.

**Fig. 3:**
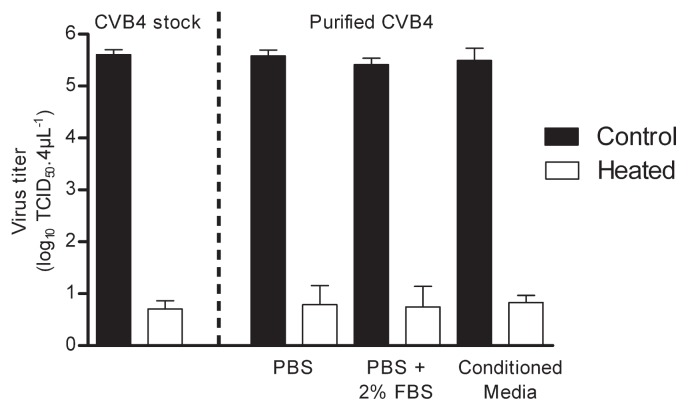
Thermal inactivation of CVB4 stock and purified CVB4 spiked in various media. 4 μL of CVB4 stock or purified CVB4 spiked (1% final dilution) in PBS or in PBS supplemented with 2% FBS or in conditioned medium enriched with cellular components obtained from mock-infected Hep-2 cell cultures were exposed (□) or not (■) to heat (70°C for 4 s). Thereafter inocula were recovered and infectious titers were determined and expressed as log_10_ TCID_50_ 4 μL^−1^ as described in legend of Fig. 1. The results are the mean ± SD of two independent experiments.

CVB4 suspension was CVB4 stock obtained from infected Hep-2 cell cultures as described in the materials and methods section, therefore the issue of the impact of cellular components and protein amounts in thermal inactivation of virus has been addressed. Experiments have been performed with CVB4 stock and purified CVB4 spiked (1% final dilution) in PBS, in PBS supplemented with 2% FBS and in conditioned medium enriched with cellular components, obtained from mock-infected Hep-2 cell cultures. When CVB4 stock and CVB4 spiked in various media was heated at 70°C for 4 s, the level of infectious particles was dramatically lower than in controls in every case (reduction ranging from 4.7 to 4.9 log_10_ reduction) (Fig 3). Thus the thermal inactivation of CVB4 stock and purified CVB4 spiked in various media in absence or in presence of protein and cellular components was similar.

## Discussion

This study is different in many respects from those of other investigators. Several considerations for the system used in the present report are noteworthy. For the first time, the level of a small volume (4 μL) of virus suspension applied on a surface subjected to heat for a short time (as short as 1 s) has been investigated. It was decided to work with 4 μL volumes for two reasons: this volume is small enough for rapid thermic transfer and large enough to contain up to 4.5 log_10_ TCID_50_ 4 μL^−1^ of infectious virus without the need to concentrate viral preparations. The individual variation of thermal resistance from one virus to a another one was unlikely due to pH of virus suspension since pH values were closely similar ranging from 7.5 to 8 (data not shown). The fast thermal inactivation in our experiments was not related to the presence of proteins or cellular components in virus suspension as illustrated in the case of purified CBV4 selected as the testing agent spiked in various media.

A critical issue was the ability to use the system at high temperature for a short time. Our system opens up the possibility to test the virucidal effect of heat under dried conditions from room temperature to 171°C which is the temperature of glycerol decomposition. One advantage of this system is that inocula immediately reached for setting temperature for the desired time and is instantly cooled at 4°C. Moreover, the system can be easily settled into a class II biological safety cabinet to prevent contamination.

Metals, like silver, iron, copper, have proven to exert antiviral activity against viruses (7, 13, 16). This study shows that conditioned medium obtained after heating aluminium foil has no virucidal effect or cell toxicity, which indicates that aluminium did not interfere in our tests. In addition, experiments combining on the one hand the measurement of infectious titers and on the other hand the estimation of the levels of viral particles through the amount of viral RNA measured by RT real-time PCR, proved the efficiency of virus recovering from aluminium foil after heat treatment.

The thermal inactivation of viruses was obtained in a very short time in most or our experiments and the inactivation curves were roughly linear, therefore, they were analyzed according to the first order kinetics model which enabled a comparison of inactivation rates obtained in our study with those obtained by other teams (24). However, first order kinetics model did not fit the inactivation curves of HSV-1 and CVB4 at 70°C and MVM at 80°C and 100°C that showed so-called shoulders and/or tails and/or downward and upward concavities (5).

Inactivation rates of H1N1 at 70 and 80°C were respectively −0.30 and −0.76 log_10_ TCID_50_ s^−1^ in our study, whereas the inactivation rates were −0.0092 and −0.023 log_10_ TCID_50_ s^−1^ at 70 and 80°C respectively in a previous study (13). The discrepancy can be explained by thermal transfer efficiency. Indeed, in our system, the virus preparation reached immediately for desired temperatures because only 4 μL were heated on a prewarmed surface whereas in the previous study a larger volume of not prewarmed liquid containing the virus (4 mL of viral preparation resuspended in 36 mL of culture media) was heated in a 50 mL polypropylene conical tube that was not prewarmed.

Although, it is commonly accepted that non-enveloped viruses are more resistant to inactivation than enveloped virus, it has been observed that, CVB4 a non-enveloped virus, was inactivated with kinetics roughly similar to those of HSV1, an enveloped virus, and that H1N1, another enveloped virus, was more resistant to heat than CVB4. One possible explanation is that heating, at relatively low temperature, provokes the release of infectious RNA of CVB4 capsid. This was already described on other viruses belonging to the Picornaviridae family which were heated to a lesser extent (37°C) (17, 23).

In the present study, for the first time, the inactivation of viral particles contained in drops exposed to temperatures higher than 100°C has been investigated. Thermal inactivation rates at such temperatures were determined with H1N1 and MVM only since HSV1 and CVB4 were fully inactivated in 1 s at 100°C. The inactivation rate of H1N1 at 110°C was −3.54 log_10_ TCID_50_ 4 μL^−1^ s^−1^, and in the case of MVM at 110, 120, and 130°C the values were −0.66, −1.54 and −2.54 log_10_ TCID_50_ 4 μL^−1^ s^−1^ respectively.

A full inactivation of MVM was obtained in our experiments when viral particles were exposed to 90°C for 1 min, however in a previous study 10 min at 90°C (wet-heat) were needed to inactivate the MVM (1). In another study (11), suspensions of MVM (1 mL) heated at 100°C for 15 min were inactivated while 9 s only were needed in our conditions. According to Eterpi *et al.* no inactivation of MVM was observed after dry heat exposure at 90°C for 1 min while in our conditions MVM was fully inactivated (9). Overall, it appears that thermal inactivation of MVM was more rapidly obtained in our experiments than in those of other authors. In our study, small drops were exposed to heating; therefore they were dried during heat treatment. Whether thermal inactivation comes from dryness has been addressed. Virus titers of HSV-1, H1N1, CVB4 and MVM drops after two hours under biosafety cabinet have been evaluated. The infectious titers of HSV-1, H1N1 and CVB4 were lower than controls (up to 2.33 log_10_ reduction in the case of HSV-1), whereas no reduction of MVM infectious titers was observed (data not shown). Thus it may be suggested that drying contribute to some extent to thermal inactivation of HSV-1, H1N1 and CVB4 but not of MVM; however drying was obtained in two hours whereas the impact of heating was obtained in a few seconds in our experiments. Nevertheless, it cannot be ruled out that the combination of water evaporation and heat intervene in thermal inactivation of viruses. Whether drying combined with heating play a role, remains an open issue, future studies will be directed along this line in our laboratory.

In conclusion, the thermal resistance of viruses contained in a small volume of suspension, has been investigated and, particularly noteworthy, the impact of short time exposure to temperatures ranging from 70°C to 130°C onto virus infectivity has been determined. This is the first report of inactivation of viral particles contained in drops exposed to temperatures higher than 100°C. The thermal resistance of H1N1, compared to HSV1 another enveloped virus, and to CVB4 a non-enveloped virus has been observed. Furthermore, it has been displayed that heating can have an unexpected faster virucidal effect than previously described.
